#  Avian Influenza A(H7N2) Virus in Human Exposed to Sick Cats, New York, USA, 2016

**DOI:** 10.3201/eid2312.170798

**Published:** 2017-12

**Authors:** Atanaska Marinova-Petkova, Jen Laplante, Yunho Jang, Brian Lynch, Natosha Zanders, Marisela Rodriguez, Joyce Jones, Sharmi Thor, Erin Hodges, Juan A. De La Cruz, Jessica Belser, Hua Yang, Paul Carney, Bo Shu, LaShondra Berman, Thomas Stark, John Barnes, Fiona Havers, Patrick Yang, Susan C. Trock, Alicia Fry, Larisa Gubareva, Joseph S. Bresee, James Stevens, Demetre Daskalakis, Dakai Liu, Christopher T. Lee, Mia Kim Torchetti, Sandra Newbury, Francine Cigel, Kathy Toohey-Kurth, Kirsten St. George, David E. Wentworth, Stephen Lindstrom, C. Todd Davis

**Affiliations:** Centers for Disease Control and Prevention, Atlanta, Georgia, USA (A. Marinova-Petkova, Y. Jang, B. Lynch, N. Zanders, M. Rodriguez, J. Jones, S. Thor, E. Hodges, J.A. De La Cruz, J. Belser, H. Yang, P. Carney, B. Shu, L. Berman, T. Stark, J. Barnes, F. Havers, P. Yang, S.C. Trock, A. Fry, L. Gubareva, J.S. Bresee, J. Stevens, D.E. Wentworth, S. Lindstrom, C.T. Davis);; New York State Department of Health, Albany, New York, USA (J. Laplante, K. St. George);; New York City Department of Health and Mental Hygiene, Long Island City, New York, USA (D. Daskalakis, D. Liu, C.T. Lee);; US Department of Agriculture, Ames, Iowa, USA (M.K. Torchetti);; University of Wisconsin, Madison, Wisconsin, USA (S. Newbury, F. Cigel, K. Toohey-Kurth)

**Keywords:** Avian influenza, A(H7N2), cat-to-human transmission, interspecies transmission, animal shelter, human infection with H7N2, New York, viruses, influenza, United States, H7N2, respiratory infections

## Abstract

An outbreak of influenza A(H7N2) virus in cats in a shelter in New York, NY, USA, resulted in zoonotic transmission. Virus isolated from the infected human was closely related to virus isolated from a cat; both were related to low pathogenicity avian influenza A(H7N2) viruses detected in the United States during the early 2000s.

Avian influenza viruses occasionally cross the species barrier, infecting humans and other mammals after exposure to infected birds and contaminated environments. Unique among the avian influenza A subtypes, both low pathogencity and highly pathogenic H7 viruses have demonstrated the ability to infect and cause disease in humans (*1**,**2*). In the eastern and northeastern United States, low pathogenicity avian influenza (LPAI) A(H7N2) viruses circulated in live bird markets periodically during 1994–2006 (*3*) and caused poultry outbreaks in Virginia, West Virginia, and North Carolina in 2002 (*4*). During an outbreak in Virginia in 2002, human infection with H7N2 virus was serologically confirmed in a culler with respiratory symptoms (*5*). In 2003, another human case of H7N2 infection was reported in a New York resident (*6*); although the source of exposure remains unknown, the isolated virus was closely related to viruses detected in live bird markets in the region. Because of the sporadic nature of these and other zoonotic infections with influenza H7 viruses throughout the world, the World Health Organization (WHO) recommended development of several candidate vaccine viruses for pandemic preparedness purposes, including 2 vaccines derived from North American lineage LPAI viruses, A/turkey/Virginia/4529/2002 and A/New York/107/2003 (*7*).

## The Study

On December 19, 2016, the New York City Department of Health and Mental Hygiene collected a respiratory specimen from a veterinarian experiencing influenza-like illness after exposure to sick domestic cats at an animal shelter in New York, NY, USA. The specimen tested positive for influenza A but could not be subtyped. Specimen aliquots were shipped to the Wadsworth Center, New York State Department of Health (Albany, NY, USA), and to the Centers for Disease Control and Prevention (CDC; Atlanta, GA, USA). Next-generation sequencing performed at the New York State Department of Health generated a partial genomic sequence (6 of 8 influenza A virus gene segments) that aligned most closely with North American lineage LPAI A(H7N2) viruses. North American lineage H7 real-time reverse transcription PCR (rRT-PCR) testing and diagnostic sequence analysis performed at CDC confirmed the sample to be positive for influenza A(H7N2) virus. Virus isolation was attempted by inoculating the sample in 10-day-old embryonated chicken eggs and MDCK CCL-34 and CRFK (Crandell-Rees Feline Kidney) cell lines (American Type Culture Collection). A/New York/108/2016 was successfully isolated from eggs but not from MDCK or CRFK cells. Codon complete sequencing of the egg-isolated virus (GISAID accession nos. EPI944622–9; http://www.gisaid.org) showed no nucleotide changes compared with the hemagglutinin (HA) and neuraminidase (NA) gene segments sequenced directly from the clinical specimen. The virus was nearly identical (99.9%) to a virus isolated from a cat, A/feline/New York/16-040082-1/2016, from a New York shelter where the veterinarian had worked; the cat died of its illness. Phylogenetic analysis of the cat and human viruses showed that their genomes were closely related to LPAI A(H7N2) viruses that were circulating in the northeastern United States in the early 2000s (Technical Appendix Figure).

Analysis of the HA gene segments revealed that A/New York/108/2016 and A/feline/New York/16-040082-1/2016 were phylogenetically related to H7N2 viruses isolated from poultry in the eastern United States (New York, Virginia, Pennsylvania, North Carolina, Massachusetts) during 1996–2005, including 2 influenza A(H7N2) WHO-recommended candidate vaccine viruses. Although the internal protein coding gene segments (polybasic 1 and 2, polyacidic, nucleoprotein, matrix, nonstructural) were distant to sequences available in databases (average nucleotide identity to the closest genetic relative was 97.6%), analysis indicated that they were of LPAI virus origin and lacked known mammalian adaptive substitutions. The longer branch lengths of the internal protein coding gene segments highlighted the scarcity of sequence data available for contemporary H7N2 viruses in the United States.

**Figure 1:**
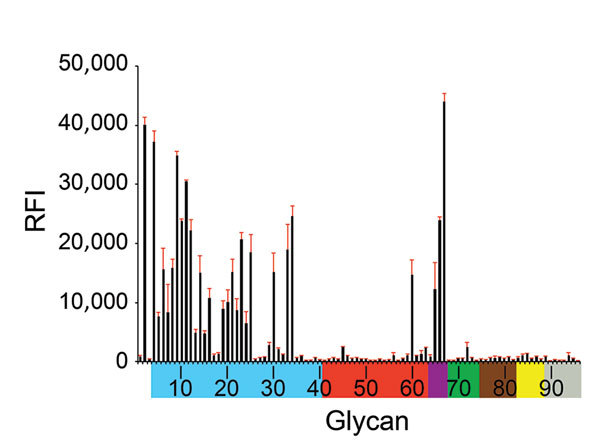
Receptor binding specificity of A/New York/108/2016 (H7N2) influenza virus isolated from a human who experienced influenza-like illness after exposure to sick domestic cats at an animal shelter in New York, NY, USA, 2016. Figure indicates glycan microarray analysis. Colored bars represent glycans that contain α-2,3 sialic acid (SA) (blue), α-2,6 SA (red), α-2,3/α-2,6 mixed SA (purple), N-glycolyl SA (green), α-2,8 SA (brown), β-2,6 and 9-O-acetyl SA (yellow), and non-SA (gray). Error bars reflect SE in the signal for 6 independent replicates on the array. RFI, relative fluorescence intensity.

Similar to well-characterized H7N2 viruses, such as A/turkey/Virginia/4529/2002 and A/New York/107/2003, A/New York/108/2016 had deletion of amino acids 212–219 in the mature HA protein (H7 numbering), known as the 220-loop of the HA receptor binding domain (*8*). Such deletion has been previously shown to enhance binding and infectivity of H7 viruses to the mammalian respiratory tract and increase direct contact transmission between mammals (*9*). Glycan microarray analysis showed that A/New York/108/2016 bound preferentially to α-2,3 avian-like receptors but also showed binding to the α-2,6 glycan with internal sialoside (LSTb, glycan #60), as well as to glycans with mixed α-2,3/α-2,6 receptors (Figure 1). Strong binding to the LSTb glycan has been previously reported for North America H7N2 viruses of avian origin (*8**,**9*) and 2013 human H7N9 viruses (*10*). The role of the LSTb glycan binding remains unknown; it has been identified only in human milk (*11*).

**Figure 2:**
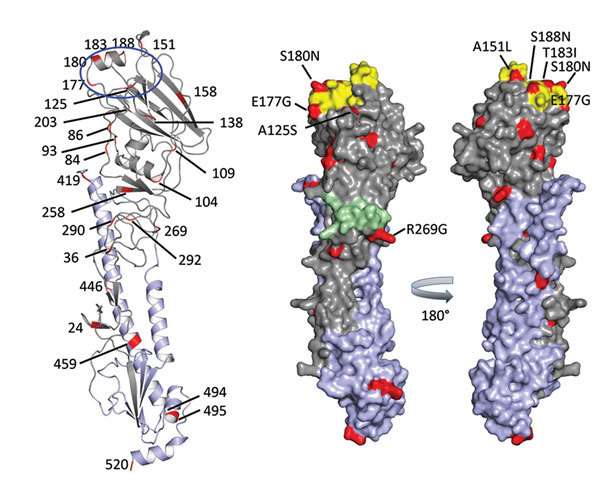
Receptor binding specificity of A/New York/108/2016 (H7N2) influenza virus isolated from a human who experienced influenza-like illness after exposure to sick domestic cats at an animal shelter in New York, NY, USA, 2016. Figure shows A/New York/108/2016 hemagglutinin (HA) monomer structure. HA1 is shown in gray, HA2 in light purple, amino acid changes in comparison with reference virus A/turkey/Virginia/4529/2002 (H7N2) in red. On the cartoon view (left), all amino acid changes in the HA protein are labeled. The location of the receptor binding site (blue circle) includes the 120-loop and the 180-helix. The 220-loop is missing due to deletion of amino acids 212–219 in the mature HA protein (H7 numbering). On the surface model (right), only amino acid substitutions adjacent to the antigenic sites and receptor binding site are labeled. Antigenic site B is yellow, antigenic site C green.

Additional molecular characterization of the HA1 protein showed 20 aa differences between A/New York/108/2016 and A/turkey/Virginia/4529/2002 (26 aa in both HA1 and HA2; Figure 2). The substitution A125S resulted in a gain of glycosylation in the HA protein of A/New York/108/2016, previously correlated with increased replication efficiency and wider tissue distribution of A/Netherlands/219/2003 (H7N7) (*12*). The substitution of T183I was shown in other avian influenza viruses (e.g., H5N1) to enhance binding to mammalian sialic acid receptors (*13*). Four of the 20 aa changes were in residues associated with antibody recognition at antigenic site B (E177G, S180N, T183I, and S188N) and antigenic site C (R269G). 

To determine the effect of these differences on antigenicity, we assessed the relationships in a 2-way hemagglutination inhibition assay, using a panel of ferret antisera raised to related H7 viruses (Table). The results showed that A/New York/108/2016 and A/feline/New York/16-040082-1/2016 reacted with α-A/turkey/Virginia/4529/2002 postinfection ferret antiserum (2-fold reduction of the hemagglutination inhibition titer compared with the A/turkey/Virginia/4529/2002 homologous titer) and α-A/New York/107/2003 antiserum (8-fold reduction compared with the A/New York/107/2003 homologous titer). These data suggest that the A/turkey/Virginia/4529/2002 candidate vaccine virus would provide cross-protection if vaccination against the 2016 H7N2 viruses was needed. Both A/turkey/Virginia/4529/2002 and A/New York/107/2003, however, reacted poorly with the antiserum raised against A/New York/108/2016.

**Table:** Hemagglutination inhibition testing of influenza A(H7) virus isolated from cat and human in New York, NY, USA, 2016, and reference viruses*

Antigens	Subtype	Ferret antisera					
		α-Gs/NE	α-Tk/MN	α-Tk/VA	α-NY/107	α-NY/108	Normal ferret serum
Reference							
A/goose/Nebraska/17097-4/11	H7N9	160	80	160	80	<10	<10
A/turkey/Minnesota/0141354/09	H7N9	20	80	20	20	<10	<10
A/turkey/Virginia/4529/02	H7N2	40	10	160	640	10	<10
A/New York/107/03	H7N2	40	20	160	640	10	<10
A/New York/108/16†	H7N2	40	10	80	80	320	<10
Test							
A/feline/New York/16-040082-1/16	H7N2	40	10	80	80	320	<10

*Gray shading indicates homologous titers. α-, reference antiserum. GS/NE, A/goose/Nebraska/17097/-4/11; Tk/MN, A/turkey/Minnesota/0141354/09; Tk/VA, A/turkey/Virginia/4529/02; NY/107, A/New York/107/03; NY/108, A/New York/108/16. †Virus isolated from human (veterinarian who experienced influenza-like illness after exposure to sick domestic cats at an animal shelter).

A 20-aa deletion in the NA stalk region, considered a genetic marker of poultry-adapted viruses (*14*), was also identified in the human and feline H7N2 viruses. No genetic markers known to reduce susceptibility to the NA inhibitor class of antiviral drugs were identified in the NA gene. Results of the NA inhibition assay indicated that the H7N2 viruses were susceptible to 4 NA inhibitors: oseltamivir, zanamivir, peramivir, and laninamivir (data not shown).

## Conclusions

The circulation of an influenza A(H7N2) virus at the animal–human interface, especially among common companion animals such as domestic cats, is of public health concern. Moreover, from an epidemiologic perspective, it is essential to understand the current distribution of LPAI A(H7N2) viruses in both avian and feline hosts. The US Department of Agriculture and state departments of agriculture have conducted routine avian influenza surveillance in live bird markets; 132,000–212,000 tests for avian influenza were performed annually during 2007–2014 (*15*), but LPAI A(H7N2) viruses were not detected. The acquisition of many genetic changes throughout the genome of the human and cat H7N2 viruses we report, however, suggests onward evolution of the virus since it was last detected in poultry and wild birds. We found that the human virus bound to α-2,6–linked sialic acid receptors, which are more common in mammals, yet retained α-2,3–linked sialic acid binding, indicating that it has dual receptor specificity; this information can be used in pandemic risk assessment of zoonotic viruses. Although human infections with LPAI A(H7N2) viruses have occurred previously, we know of no other reported instances of direct transmission from a cat to a human.

Technical Appendix Description of methods used in the study of avian influenza A(H7N2) and neighbor-joining phylogenetic trees of various internal genes.
