# Supplementary material for: Avian Influenza A(H7N2) Virus in Human Exposed to Sick Cats, New York, USA, 2016
Source: Emerg Infect Dis. 2017 Dec;23(12):2046–9. doi: 10.3201/eid2312.170798 (PMC5708219; doi:10.3201/eid2312.170798)
Supplement: Technical Appendix — Description of methods used in the study of avian influenza A(H7N2) and neighbor-joining phylogenetic trees of various internal genes. [file 17-0798-Techapp-s1.pdf]

Article DOI: https://doi.org/10.3201/eid2312.170798

Avian Influenza A(H7N2) Virus in Human Exposed to Sick Cats, New York, USA, 2016

Technical Appendix

Methods

Influenza A Confirmation and Subtyping of the Clinical Specimen

RNA was extracted from the clinical specimen using Qiamp viral RNA purification kit (QIAGEN, Hilden, Germany) at Wadsworth Center, and MagNA Pure Compact Nucleic Acid Isolation Kit I (Roche Diagnostics Corporation, Indianapolis, IN, USA), at the Centers for Disease Control and Prevention (CDC). Influenza A virus was confirmed by using rRT-PCR with primers and probe for detection of universal influenza A Matrix gene (CDC Laboratory Support for Influenza Surveillance, Centers for Disease Control and Prevention, Atlanta, GA, USA); and subtyped using specific primers and probe for North American lineage influenza A(H7) viruses (primer and probe sequences available upon request). The A(H7N2) subtype was further confirmed by Sanger sequencing analysis of PCR products amplified using a single step RT-PCR reaction with H7 and N2-specific primers (available upon request). Illumina next-generation sequencing of the viral RNA was performed using MiSeq analysis (Illumina, San Diego, CA, USA) and sequence data was analyzed with IRMA (*1*).

Virus isolation

Virus isolation was attempted in Madin Darby Canine Kidney (MDCK) and Crandell-Rees Feline Kidney (CRFK) cell lines (ATCC), and in 10-day-old embryonated chicken eggs (*2*). Cell cultures were incubated at 35°C for 72 hours and checked for cytopathic effect twice daily. Inoculated eggs were chilled after 48 hours of incubation at 35°C. Presence of influenza A virus in the allantoic fluids of eggs, but not cell-culture supernatants, was confirmed by positive reaction hemagglutination with 0.5% suspension of turkey erythrocytes.

Viral Genome Sequencing, Phylogenetic Analysis, and HA Monomer Protein Structure Modeling

Codon complete genome sequencing of virus isolated in embryonated chicken eggs was performed using MiSeq analysis. Gene sequences were submitted to GISAID with the following accession numbers: PB2, EPI944626; PB1, EPI944627; PA, EPI944625; HA, EPI944629; NP, EPI944622; NA, EPI944628; MP, EPI944624; and NS, EPI944623. Reference sequences for the phylogenetic reconstruction were retrieved from the GenBank and GISAID databases (*3*,*4*). Codon complete genome sequences were aligned via MUSCLE (*5*) and HA sequences were trimmed to the start of the mature H7 HA protein sequence using BioEdit v7.0 (*6*). Neighbor-joining phylogenetic trees (Jukes-Cantor model) with 1,000 bootstrap replicates were constructed using MEGA 5.05 (*7*). The model of HA monomer structure was generated using SWISS – Model (*8*) with 3M5G as the starting model. All the structural figures were generated using MacPyMOL (*9*).

Antigenic Characterization

Hemagglutination inhibition (HI) testing was performed by using selected subtype H7 WHO candidate vaccine viruses and CDC reference viruses of the North American lineage, as well as postinfection ferret antisera produced against H7 viruses of the North American lineage (see Table in main article). Turkey erythrocytes at 0.5% concentration were used for the HI test (*10*). All antisera used in the HI test were treated with receptor-destroying enzyme (Denka Seiken, Tokyo, Japan) according to the manufacturer’s recommendations, and used at 1:10 starting dilution.

Glycan Microarray Analysis

Glycan microarray slides were produced under contract for CDC using a glycan library generously provided by the Consortium for Functional Glycomics (www.functionalglycomics.org), funded by National Institute of General Medical Sciences grant GM62116 (Technical Appendix Table, glycans used for analyses in these experiments). Virus preparations were diluted in phosphate-buffered saline (PBS) with 2% (wt/vol) bovine serum albumin to an HA titer of 128. Virus suspensions were applied to the slides, and the slides were incubated in a closed container (at 4°C) subjected to gentle agitation for 1.5 hours. Unbound virus was washed off with brief sequential rinses in PBS with 0.05% Tween 20 (PBS-T) and PBS. The slides were then immediately incubated with ferret serum raised against A/New York/108/2016 (30 min); a biotinylated anti-ferret IgG antibody (Rockland) in combination with

streptavidin-Alexa Fluor488 conjugate (30 min) (Thermo Fisher, Waltham, MA, USA), with brief PBS-T/PBS washes being performed after each incubation. After the final PBS–T/PBS washes, the slides were washed briefly in deionized water, dried by a gentle steam of nitrogen gas, and immediately subjected to imaging. Fluorescence intensities were detected using an Innoscan 1100AL scanner (Innopsys, Carbonne, France). Image analyses were carried out using ImaGene 9 image analysis software (BioDiscovery, El Segundo, CA, USA).

References

1. Shepard SS, Meno S, Bahl J, Wilson MM, Barnes J, Neuhaus E. Viral deep sequencing needs an adaptive approach: IRMA, the iterative refinement meta-assembler. BMC Genomics. 2016;17:708. http://dx.doi.org/10.1186/s12864-016-3030-6

2. Shortridge KF, Butterfield WK, Webster RG, Campbell CH. Isolation and characterization of influenza A viruses from avian species in Hong Kong. Bull World Health Organ. 1977;55:15–20.

3. Benson DA, Karsch-Mizrachi I, Lipman DJ, Ostell J, Wheeler DL. GenBank. Nucleic Acids Res. 2005;33:D34–8. http://dx.doi.org/10.1093/nar/gki063

4. Bogner P, Capua I, Lipman DJ, Cox NJ, et al. A global initiative on sharing avian flu data. Nature. 2006;442:981 http://platform.gisaid.org. http://dx.doi.org/10.1038/442981a

5. Edgar RC. MUSCLE: multiple sequence alignment with high accuracy and high throughput. Nucleic Acids Res. 2004;32:1792–7. http://dx.doi.org/10.1093/nar/gkh340

6. Hall TA. BioEdit: A user-friendly biological sequence alignment editor and analysis program for Windows 95/98/NT. Nucleic Acids Symp Ser. 1999;41:95–8.

7. Tamura K, Peterson D, Peterson N, Stecher G, Nei M, Kumar S. MEGA5: molecular evolutionary genetics analysis using maximum likelihood, evolutionary distance, and maximum parsimony methods. Mol Biol Evol. 2011;28:2731–9. http://dx.doi.org/10.1093/molbev/msr121

8. Biasini M, Bienert S, Waterhouse A, Arnold K, Studer G, Schmidt T, et al. SWISS-MODEL: modelling protein tertiary and quaternary structure using evolutionary information. Nucleic Acids Res. 2014;42(W1):W252-8. http://dx.doi.org/10.1093/nar/gku340

9. DeLano WL. The PyMol Molecular Graphics Systems. 2002. [cited 2017 May 5]. www.pymol.org

10. Palmer DFDW, Coleman MT, Schild GC. Advanced laboratory techniques for influenza diagnosis. Washington, DC: US Department of Health, Education and Welfare, 1975.

Technical Appendix Table. Glycan microarray for H7N2 viruses*

No.	Structure
1	Neu5Acα
2	Neu5Acα
3	Neu5Acβ
4	Neu5Acα2–3(6-O-Su)Galβ1–4GlcNAcβ
5	Neu5Acα2–3Galβ1–3[6OSO3]GalNAcα
6	Neu5Acα2–3Galβ1–4[6OSO3]GlcNAcβ
7	Neu5Acα2–3Galβ1–4(Fucα1–3)[6OSO3]GlcNAcβ-propyl-NH2
8	Neu5Acα2–3Galβ1–3[6OSO3]GlcNAc-propyl-NH2
9	Neu5Acα2–3Galβ1–3(Neu5Acα2–3Galβ1–4)GlcNAcβ
10	Neu5Acα2–3Galβ1–3(Neu5Acα2–3Galβ1–4GlcNAcβ1–6)GalNAcα
11	Neu5Acα2–3Galβ1–4GlcNAcβ1–2Manα1–3(Neu5Acα2–3Galβ1–4GlcNAcβ1–2Manα1–6)Manβ1–4GlcNAcβ1–4GlcNAcβ
12	Neu5Acα(2–3)-Galβ(1–4)-GlcNAcβ(1–3)-Galβ(1–4)-GlcNAcβ(1–2)-Manα(1–3)-[Neu5Acα(2–3)-Galβ(1–4)-GlcNAcβ(1–3)-Galβ(1–4)-GlcNAcβ(1–2)-Manα(1–6)]-Manβ(1–4)-GlcNAcβ(1–4)-GlcNAcβ
13	Neu5Acα2–3Galβ
14	Neu5Acα2–3Galβ1–3GalNAcα
15	Neu5Acα2–3Galβ1–3GlcNAcβ
16	Neu5Acα2–3Galβ1–3GlcNAcβ
17	Neu5Acα2–3Galβ1–4Glcβ
18	Neu5Acα2–3Galβ1–4Glcβ
19	Neu5Acα2–3Galβ1–4GlcNAcβ
20	Neu5Acα2–3Galβ1–4GlcNAcβ
21	Neu5Acα2–3GalNAcβ1–4GlcNAcβ
22	Neu5Acα2–3Galβ1–4GlcNAcβ1–3Galβ1–4GlcNAcβ
23	Neu5Aca2–3Galβ1–3GlcNAcβ1–3Galβ1–4GlcNAcβ
24	Neu5Acα2–3Galβ1–4GlcNAcβ1–3Galβ1–4GlcNAcβ1–3Galβ1–4GlcNAcβ
25	Neu5Acα2–3Galβ1–4GlcNAcβ1–3Galβ1–3GlcNAcβ
26	Neu5Acα2–3Galβ1–3GalNAcα
27	Galβ1–3(Neu5Acα2–3Galβ1–4(Fucα1–3)GlcNAcβ1–6)GalNAcα
28	Neu5Acα2–3Galβ1–3(Fucα1–4)GlcNAcβ
29	Neu5Acα2–3Galβ1–4(Fucα1–3)GlcNAcβ
30	Neu5Acα2–3Galβ1–4(Fucα1–3)GlcNAcβ
31	Neu5Acα2–3Galβ1–4(Fucα1–3)GlcNAcβ1–3Galβ
32	Neu5Acα2–3Galβ1–3[Fucα1–4]GlcNAcβ1–3Galβ1–4[Fucα1–3]GlcNAcβ
33	Neu5Acα2–3Galβ1–3[Fucα1–3]GlcNAcβ1–3Galβ1–4[Fucα1–3]GlcNAcβ
34	Neu5Acα2–3Galβ1–4(Fucα1–3)GlcNAcβ1–3Galβ1–4(Fucα1–3)GlcNAcβ1–3Galβ1–4(Fucα1–3)GlcNAcβ
35	Neu5Acα2–3(GalNAcβ1–4)Galβ1–4GlcNAcβ
36	Neu5Acα2–3(GalNAcβ1–4)Galβ1–4GlcNAcβ
37	Neu5Acα2–3(GalNAcβ1–4)Galβ1–4Glcβ
38	Galβ1–3GalNAcβ1–4(Neu5Acα2–3)Galβ1–4Glcβ
39	Fucα1–2Galβ1–3GalNAcβ1–4(Neu5Acα2–3)Galβ1–4Glcβ
40	Fucα1–2Galβ1–3GalNAcβ1–4(Neu5Acα2–3)Galβ1–4Glcβ
41	Neu5Acα2–6Galβ1–4[6OSO3]GlcNAcβ
42	Neu5Acα2–6Galβ1–4GlcNAcβ1–2Manα1–3(Galβ1–4GlcNAcβ1–2Manα1–6)Manβ1–4GlcNAcβ1–4GlcNAcβ
43	Neu5Acα2–6Galβ1–4GlcNAcβ1–2Manα1–3(Neu5Acα2–6Galβ1–4GlcNAcβ1–2Manα1–6)Manβ1–4GlcNAcβ1–4GlcNAcβ
44	Neu5Acα2–6Galβ1–4GlcNAcβ1–3Galβ1–4GlcNAcβ1–2Manα1–3[Neu5Acα2–6Galβ1–4GlcNAcβ1–3Galβ1–4GlcNAcβ1–2Manα1–6]Manβ1–4GlcNAcβ1–4GlcNAcβ
45	Neu5Acα2–6Galβ1–4GlcNAcβ1–3Galβ1–4GlcNAcβ1–3Galβ1–4GlcNAcβ1–2Manα1–3[Neu5Acα2–6Galβ1–4GlcNAcβ1–3Galβ1–4GlcNAcβ1–3Galβ1–4GlcNAcβ1–2Manα1–6]-Manβ1–4GlcNAcβ1–4GlcNAcβ
46	Neu5Acα2–6Galβ1–4GlcNAcβ1–3Galβ1–4GlcNAcβ1–3[Neu5Acα2–6Galβ1–4GlcNAcβ1–3Galβ1–4GlcNAcβ1–6]GalNAca
47	Neu5Acα2–6Galβ1–4GlcNAcβ1–3[Neu5Acα2–6Galβ1–4GlcNAcβ1–6]GalNAca
48	Neu5Acα2–6GalNAcα
49	Neu5Acα2–6Galβ
50	Neu5Acα2–6Galβ1–4Glcβ
51	Neu5Acα2–6Galβ1–4Glcβ
52	Neu5Acα2–6Galβ1–4GlcNAcβ
53	Neu5Acα2–6Galβ1–4GlcNAcβ
54	Neu5Acα2–6GalNAcβ1–4GlcNAcβ
55	Neu5Acα2–6Galβ1–4GlcNAcβ1–3GalNAcα
56	Neu5Acα2–6Galβ1–4GlcNAcβ1–3Galβ1–4GlcNAcβ

No.	Structure
57	Neu5Acα2–6Galβ1–4GlcNAcβ1–3Galβ1–4GlcNAcβ1–3GalNAcα
58	Neu5Aca2–6Galβ1–4GlcNAcβ1–3Galβ1–4GlcNAcβ1–3Galβ1–4GlcNAcβ
59	Neu5Acα2–6Galβ1–4GlcNAcβ1–3Galβ1–4(Fucα1–3)GlcNAcβ1–3Galβ1–4(Fucα1–3)GlcNAcβ
60	Galβ1–3(Neu5Acα2–6)GlcNAcβ1–4Galβ1–4Glcβ-Sp10
61	Neu5Acα2–6[Galβ1–3]GalNAca
62	Neu5Acα2–6Galβ1–4GlcNAcβ1–6[Galβ1–3]GalNAca
63	Neu5Acα2–6Galβ1–4GlcNAcβ1–3Galβ1–4GlcNAcβ1–6[Galβ1–3]GalNAca
64	Neu5Acα2–3Galβ1–4GlcNAcβ1–2Manα1–3(Neu5Acα2–6Galβ1–4GlcNAcβ1–2Manα1–6)Manβ1–4GlcNAcβ1–4GlcNAcβ
65	Neu5Acα2–6Galβ1–4GlcNAcβ1–2Manα1–3(Neu5Acα2–3Galβ1–4GlcNAcβ1–2Manα1–6)Manβ1–4GlcNAcβ1–4GlcNAcβ
66	Neu5Acα2–3Galβ1–3(Neu5Acα2–6)GalNAcα
67	Neu5Acα2–3(Neu5Acα2–6)GalNAcα
68	Neu5Gcα
69	Neu5Gcα2–3Galβ1–3(Fucα1–4)GlcNAcβ
70	Neu5Gcα2–3Galβ1–3GlcNAcβ
71	Neu5Gcα2–3Galβ1–4(Fucα1–3)GlcNAcβ
72	Neu5Gcα2–3Galβ1–4GlcNAcβ
73	Neu5Gcα2–6GalNAcα
74	Neu5Gcα2–6Galβ1–4GlcNAcβ
75	Neu5Acα2–8Neu5Acα
76	Neu5Acα2–8Neu5Acα2–8Neu5Acα
77	Neu5Acα2–8Neu5Acα2–3(GalNAcβ1–4)Galβ1–4Glcβ
78	Neu5Acα2–8Neu5Acα2–3Galβ1–4Glcβ
79	Neu5Acα2–8Neu5Acα2–8Neu5Acα2–3(GalNAcβ1–4)Galβ1–4Glcβ
80	Neu5Acα2–8Neu5Acα2–8Neu5Acα2–3Galβ1–4Glcβ
81	Neu5Acα2–8Neu5Acβ-Sp17
82	Neu5Acα2–8Neu5Acα2–8Neu5Acβ
83	Neu5Acβ2–6GalNAcα
84	Neu5Acβ2–6Galβ1–4GlcNAcβ
85	Neu5Gcβ2–6Galβ1–4GlcNAc
86	Galβ1–3(Neu5Acβ2–6)GalNAcα
87	[9NAc]Neu5Acα
88	[9NAc]Neu5Acα2–6Galβ1–4GlcNAcβ
89	Galβ1–4GlcNAcβ1–3Galβ1–4GlcNAcβ1–3Galβ1–4GlcNAcβ
90	Galβ1–3GlcNAcβ1–3Galβ1–3GlcNAcβ
91	Galβ1–4GlcNAcβ1–2Manα1–3[Galβ1–4GlcNAcβ1–2Manα1–6]Manβ1–4GlcNAcβ1–4GlcNAcβ
92	GalNAcα1–3(Fucα1–2)Galβ1–3GlcNAcβ
93	GalNAcα1–3(Fucα1–2)Galβ1–4GlcNAcβ
94	Galα1–3(Fucα1–2)Galβ1–3GlcNAcβ
95	Galα1–3(Fucα1–2)Galβ1–4(Fucα1–3)GlcNAcβ
96	Galβ1–3GalNAcα

*Colors represent glycans that contain α-2,3 sialic acid (SA) (blue), α-2,6 SA (red), α-2,3/α-2,6 mixed SA (purple), N-glycolyl SA (green), α-2,8 SA (brown), β-2,6 and 9-O-acetyl SA (yellow), and non-SA (gray).

A

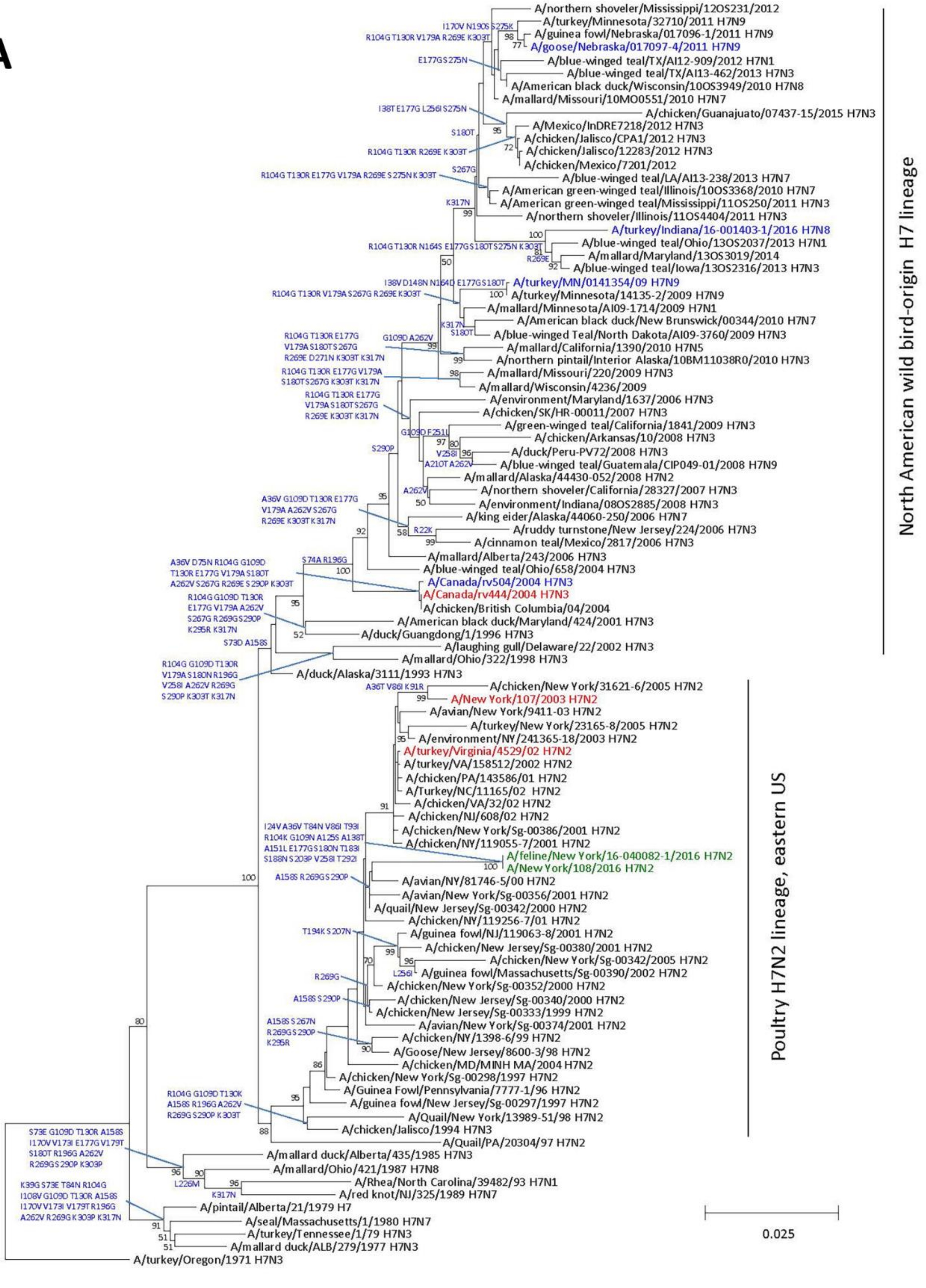
A/northern shoveler/Mississippi/12OS231/2012
A/turkey/Minnesota/32710/2011 H7N9
A/guinea fowl/Nebraska/017096-1/2011 H7N9
A/goose/Nebraska/017097-4/2011 H7N9
A/blue-winged teal/TX/AI12-909/2012 H7N1
A/blue-winged teal/TX/AI13-462/2013 H7N3
A/American black duck/Wisconsin/10OS3949/2010 H7N8
A/mallard/Missouri/10MO0551/2010 H7N7
A/chicken/Guanajuato/07437-15/2015 H7N3
A/Mexico/InDRE7218/2012 H7N3
A/chicken/Jalisco/CPA1/2012 H7N3
A/chicken/Jalisco/12283/2012 H7N3
A/chicken/Mexico/7201/2012
A/blue-winged teal/LA/AI13-238/2013 H7N7
A/American green-winged teal/Illinois/10OS3368/2010 H7N7
A/American green-winged teal/Mississippi/11OS250/2011 H7N3
A/northern shoveler/Illinois/11OS4404/2011 H7N3
A/turkey/Indiana/16-001403-1/2016 H7N8
A/blue-winged teal/Ohio/13OS2037/2013 H7N1
A/mallard/Maryland/13OS3019/2014
A/blue-winged teal/Iowa/13OS2316/2013 H7N3
A/turkey/MN/0141354/09 H7N9
A/turkey/Minnesota/14135-2/2009 H7N9
A/mallard/Minnesota/AI09-1714/2009 H7N1
A/American black duck/New Brunswick/00344/2010 H7N7
A/blue-winged Teal/North Dakota/AI09-3760/2009 H7N3
A/mallard/California/1390/2010 H7N5
A/northern pintail/Interior Alaska/10BM11038R0/2010 H7N3
A/mallard/Missouri/220/2009 H7N3
A/mallard/Wisconsin/4236/2009
A/environment/Maryland/1637/2006 H7N3
A/chicken/SK/HR-00011/2007 H7N3
A/green-winged teal/California/1841/2009 H7N3
A/chicken/Arkansas/10/2008 H7N3
A/duck/Peru-PV72/2008 H7N3
A/blue-winged teal/Guatemala/CIP049-01/2008 H7N9
A/mallard/Alaska/44430-052/2008 H7N2
A/northern shoveler/California/28327/2007 H7N3
A/environment/Indiana/08OS2885/2008 H7N3
A/king eider/Alaska/44060-250/2006 H7N7
A/ruddy turnstone/New Jersey/224/2006 H7N3
A/cinnamon teal/Mexico/2817/2006 H7N3
A/mallard/Alberta/243/2006 H7N3
A/blue-winged teal/Ohio/658/2004 H7N3
A/Canada/rv504/2004 H7N3
A/Canada/rv444/2004 H7N3
A/chicken/British Columbia/04/2004
A/American black duck/Maryland/424/2001 H7N3
A/duck/Guangdong/1/1996 H7N3
A/laughing gull/Delaware/22/2002 H7N3
A/mallard/Ohio/322/1998 H7N3
A/duck/Alaska/3111/1993 H7N3
A/chicken/New York/31621-6/2005 H7N2
A/New York/107/2003 H7N2
A/avian/New York/9411-03 H7N2
A/turkey/New York/23165-8/2005 H7N2
A/environment/NY/241365-18/2003 H7N2
A/turkey/Virginia/4529/02 H7N2
A/turkey/VA/158512/2002 H7N2
A/chicken/PA/143586/01 H7N2
A/Turkey/NC/11165/02 H7N2
A/chicken/VA/32/02 H7N2
A/chicken/NJ/608/02 H7N2
A/chicken/New York/Sg-00386/2001 H7N2
A/chicken/NY/119055-7/2001 H7N2
A/feline/New York/16-040082-1/2016 H7N2
A/New York/108/2016 H7N2
A/avian/NY/81746-5/00 H7N2
A/avian/New York/Sg-00356/2001 H7N2
A/quail/New Jersey/Sg-00342/2000 H7N2
A/chicken/NY/119256-7/01 H7N2
A/guinea fowl/NJ/119063-8/2001 H7N2
A/chicken/New Jersey/Sg-00380/2001 H7N2
A/chicken/New York/Sg-00342/2005 H7N2
A/guinea fowl/Massachusetts/Sg-00390/2002 H7N2
A/chicken/New York/Sg-00352/2000 H7N2
A/chicken/New Jersey/Sg-00340/2000 H7N2
A/chicken/New Jersey/Sg-00333/1999 H7N2
A/avian/New York/Sg-00374/2001 H7N2
A/chicken/NY/1398-6/99 H7N2
A/Goose/New Jersey/8600-3/98 H7N2
A/chicken/MD/MINH MA/2004 H7N2
A/chicken/New York/Sg-00298/1997 H7N2
A/Guinea Fowl/Pennsylvania/7777-1/96 H7N2
A/guinea fowl/New Jersey/Sg-00297/1997 H7N2
A/Quail/New York/13989-51/98 H7N2
A/chicken/Jalisco/1994 H7N3
A/Quail/PA/20304/97 H7N2
A/mallard duck/Alberta/435/1985 H7N3
A/mallard/Ohio/421/1987 H7N8
A/Rhea/North Carolina/39482/93 H7N1
A/red knot/NJ/325/1989 H7N7
A/pintail/Alberta/21/1979 H7
A/seal/Massachusetts/1/1980 H7N7
A/turkey/Tennessee/1/79 H7N3
A/mallard duck/ALB/279/1977 H7N3
A/turkey/Oregon/1971 H7N3
North American wild bird-origin H7 lineage
Poultry H7N2 lineage, eastern US
0.025
I170V N190S S275K
R104G T130R V179A R269E K303T
E177G S275N
I38T E177G L256I S275N
S180T
R104G T130R R269E K303T
S267G
R104G T130R E177G V179A R269E S275N K303T
K317N
R104G T130R N164S E177G S180T S275N K303T
R269E
I38V D148N N164D E177G S180T
R104G T130R V179A S267G R269E K303T
K317N
S180T
G109D A262V
R104G T130R E177G V179A S180T S267G R269E D271N K303T K317N
R104G T130R E177G V179A S180T S267G K303T K317N
R104G T130R E177G V179A S180T S267G R269E K303T K317N
G109D F251L
V258I
A210T A262V
S290P
A262V
A36V G109D T130R E177G V179A A262V S267G R269E K303T K317N
R22K
S74A R196G
A36V D75N R104G G109D T130R E177G V179A S180T A262V S267G R269E S290P K303T
R104G G109D T130R E177G V179A A262V S267G R269G S290P K295R K317N
S73D A158S
R104G G109D T130R V179A S180N R196G V258I A262V R269G S290P K303T K317N
A36T V86I K91R
I24V A36V T84N V86I T93I R104K G109N A125S A138T A151L E177G S180N T183I S188N S203P V258I T292I
A158S R269G S290P
T194K S207N
L256I
R269G
A158S S290P
A158S S267N R269G S290P K295R
R104G G109D T130K A158S R196G A262V R269G S290P K303T
S73E G109D T130R A158S I170V V173I E177G V179T S180T R196G A262V R269G S290P K303P
L226M
K317N
K39G S73E T84N R104G I108V G109D T130R A158S I170V V173I V179T R196G A262V R269G K303P K317N
98
77
95
72
99
100
81
92
50
100
99
99
98
97
80
96
50
95
58
99
92
100
95
52
99
95
91
100
100
99
96
70
90
86
95
88
80
96
90
96
91
51
51

B

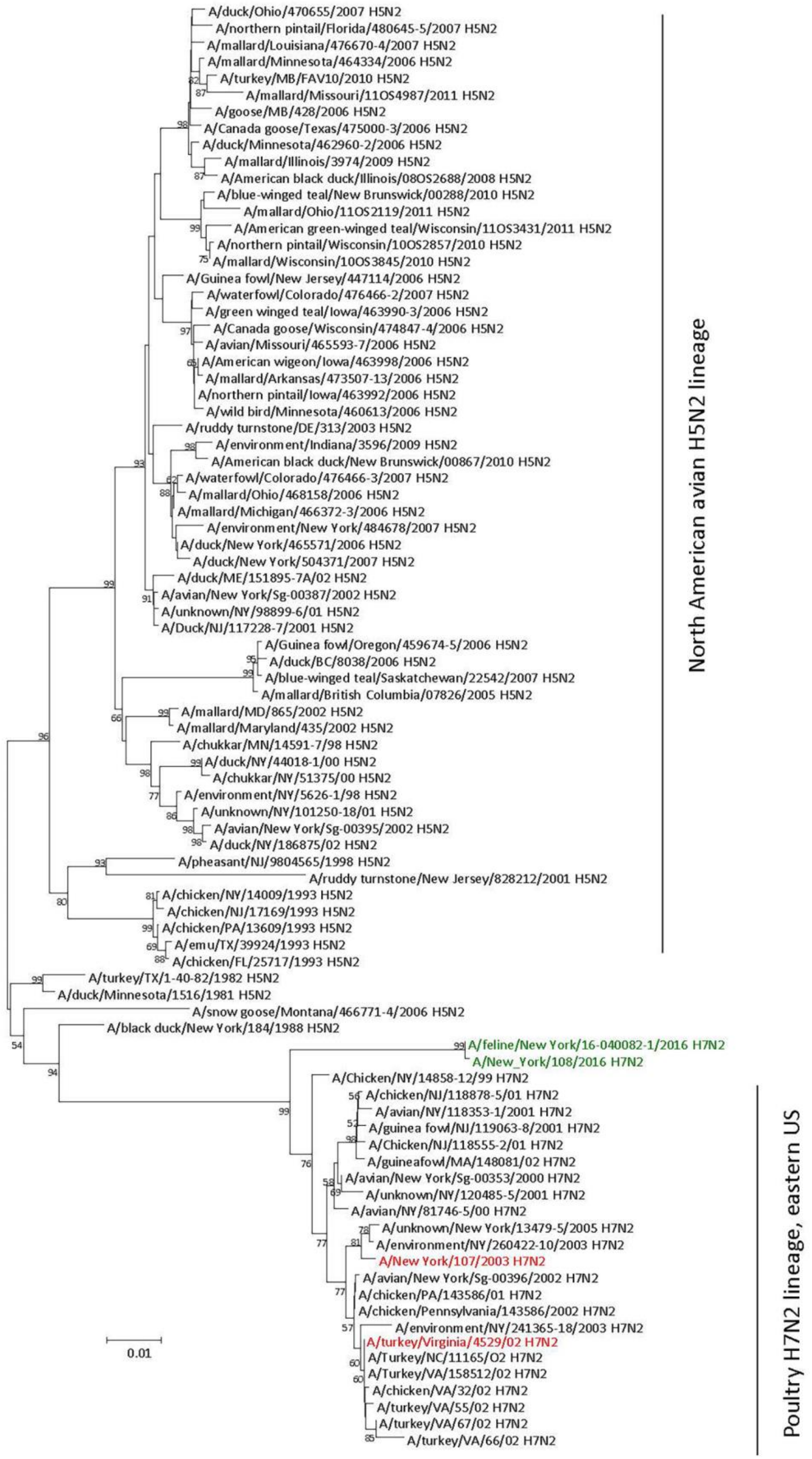
A/duck/Ohio/470655/2007 H5N2
A/northern pintail/Florida/480645-5/2007 H5N2
A/mallard/Louisiana/476670-4/2007 H5N2
A/mallard/Minnesota/464334/2006 H5N2
A/turkey/MB/FAV10/2010 H5N2
A/mallard/Missouri/11OS4987/2011 H5N2
A/goose/MB/428/2006 H5N2
A/Canada goose/Texas/475000-3/2006 H5N2
A/duck/Minnesota/462960-2/2006 H5N2
A/mallard/Illinois/3974/2009 H5N2
A/American black duck/Illinois/08OS2688/2008 H5N2
A/blue-winged teal/New Brunswick/00288/2010 H5N2
A/mallard/Ohio/11OS2119/2011 H5N2
A/American green-winged teal/Wisconsin/11OS3431/2011 H5N2
A/northern pintail/Wisconsin/10OS2857/2010 H5N2
A/mallard/Wisconsin/10OS3845/2010 H5N2
A/Guinea fowl/New Jersey/447114/2006 H5N2
A/waterfowl/Colorado/476466-2/2007 H5N2
A/green winged teal/Iowa/463990-3/2006 H5N2
A/Canada goose/Wisconsin/474847-4/2006 H5N2
A/avian/Missouri/465593-7/2006 H5N2
A/American wigeon/Iowa/463998/2006 H5N2
A/mallard/Arkansas/473507-13/2006 H5N2
A/northern pintail/Iowa/463992/2006 H5N2
A/wild bird/Minnesota/460613/2006 H5N2
A/ruddy turnstone/DE/313/2003 H5N2
A/environment/Indiana/3596/2009 H5N2
A/American black duck/New Brunswick/00867/2010 H5N2
A/waterfowl/Colorado/476466-3/2007 H5N2
A/mallard/Ohio/468158/2006 H5N2
A/mallard/Michigan/466372-3/2006 H5N2
A/environment/New York/484678/2007 H5N2
A/duck/New York/465571/2006 H5N2
A/duck/New York/504371/2007 H5N2
A/duck/ME/151895-7A/02 H5N2
A/avian/New York/Sg-00387/2002 H5N2
A/unknown/NY/98899-6/01 H5N2
A/Duck/NJ/117228-7/2001 H5N2
A/Guinea fowl/Oregon/459674-5/2006 H5N2
A/duck/BC/8038/2006 H5N2
A/blue-winged teal/Saskatchewan/22542/2007 H5N2
A/mallard/British Columbia/07826/2005 H5N2
A/mallard/MD/865/2002 H5N2
A/mallard/Maryland/435/2002 H5N2
A/chukkar/MN/14591-7/98 H5N2
A/duck/NY/44018-1/00 H5N2
A/chukkar/NY/51375/00 H5N2
A/environment/NY/5626-1/98 H5N2
A/unknown/NY/101250-18/01 H5N2
A/avian/New York/Sg-00395/2002 H5N2
A/duck/NY/186875/02 H5N2
A/pheasant/NJ/9804565/1998 H5N2
A/ruddy turnstone/New Jersey/828212/2001 H5N2
A/chicken/NY/14009/1993 H5N2
A/chicken/NJ/17169/1993 H5N2
A/chicken/PA/13609/1993 H5N2
A/emu/TX/39924/1993 H5N2
A/chicken/FL/25717/1993 H5N2
A/turkey/TX/1-40-82/1982 H5N2
A/duck/Minnesota/1516/1981 H5N2
A/snow goose/Montana/466771-4/2006 H5N2
A/black duck/New York/184/1988 H5N2
A/feline/New York/16-040082-1/2016 H7N2
A/New_York/108/2016 H7N2
A/Chicken/NY/14858-12/99 H7N2
A/chicken/NJ/118878-5/01 H7N2
A/avian/NY/118353-1/2001 H7N2
A/guinea fowl/NJ/119063-8/2001 H7N2
A/Chicken/NJ/118555-2/01 H7N2
A/guineafowl/MA/148081/02 H7N2
A/avian/New York/Sg-00353/2000 H7N2
A/unknown/NY/120485-5/2001 H7N2
A/avian/NY/81746-5/00 H7N2
A/unknown/New York/13479-5/2005 H7N2
A/environment/NY/260422-10/2003 H7N2
A/New York/107/2003 H7N2
A/avian/New York/Sg-00396/2002 H7N2
A/chicken/PA/143586/01 H7N2
A/chicken/Pennsylvania/143586/2002 H7N2
A/environment/NY/241365-18/2003 H7N2
A/turkey/Virginia/4529/02 H7N2
A/Turkey/NC/11165/O2 H7N2
A/Turkey/VA/158512/02 H7N2
A/chicken/VA/32/02 H7N2
A/turkey/VA/55/02 H7N2
A/turkey/VA/67/02 H7N2
A/turkey/VA/66/02 H7N2
North American avian H5N2 lineage
Poultry H7N2 lineage, eastern US
0.01

C

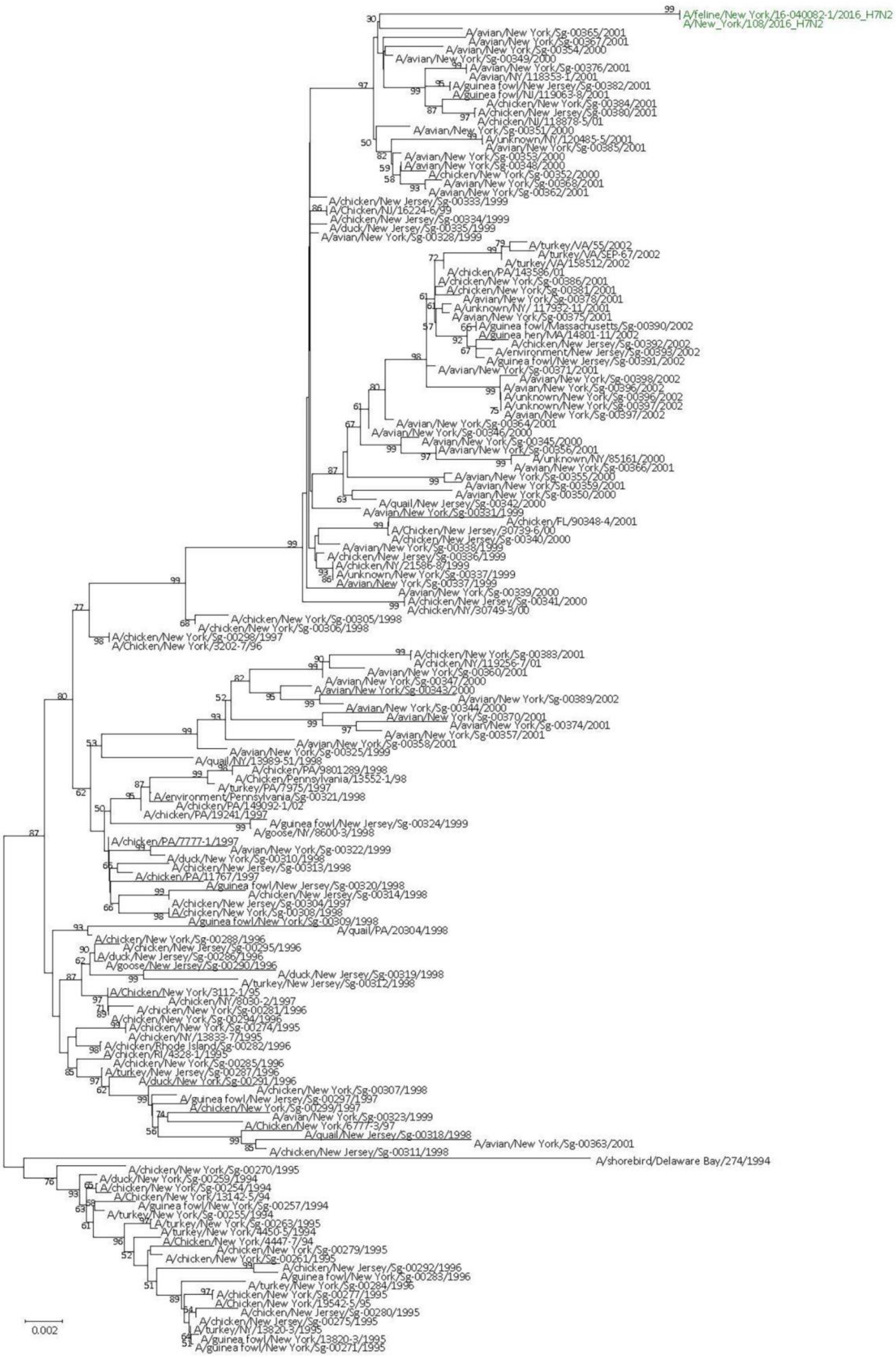
A/feline/New York/16-040082-1/2016_H7N2
A/New_York/108/2016_H7N2
A/avian/New York/Sg-00365/2001
A/avian/New York/Sg-00367/2001
A/avian/New York/Sg-00354/2000
A/avian/New York/Sg-00349/2000
A/avian/New York/Sg-00376/2001
A/avian/NY/118353-1/2001
A/guinea fowl/New Jersey/Sg-00382/2001
A/guinea fowl/NJ/119063-8/2001
A/chicken/New York/Sg-00384/2001
A/chicken/New Jersey/Sg-00380/2001
A/chicken/NJ/118878-5/01
A/avian/New York/Sg-00351/2000
A/unknown/NY/120485-5/2001
A/avian/New York/Sg-00385/2001
A/avian/New York/Sg-00353/2000
A/avian/New York/Sg-00348/2000
A/chicken/New York/Sg-00352/2000
A/avian/New York/Sg-00368/2001
A/avian/New York/Sg-00362/2001
A/chicken/New Jersey/Sg-00333/1999
A/Chicken/NJ/16224-6/99
A/chicken/New Jersey/Sg-00334/1999
A/duck/New Jersey/Sg-00335/1999
A/avian/New York/Sg-00328/1999
A/turkey/VA/55/2002
A/turkey/VA/SEP-67/2002
A/turkey/VA/158512/2002
A/chicken/PA/143586/01
A/chicken/New York/Sg-00386/2001
A/chicken/New York/Sg-00381/2001
A/avian/New York/Sg-00378/2001
A/unknown/NY/ 117932-11/2001
A/avian/New York/Sg-00375/2001
A/guinea fowl/Massachusetts/Sg-00390/2002
A/guinea hen/MA/14801-11/2002
A/chicken/New Jersey/Sg-00392/2002
A/environment/New Jersey/Sg-00393/2002
A/guinea fowl/New Jersey/Sg-00391/2002
A/avian/New York/Sg-00371/2001
A/avian/New York/Sg-00398/2002
A/avian/New York/Sg-00396/2002
A/unknown/New York/Sg-00396/2002
A/unknown/New York/Sg-00397/2002
A/avian/New York/Sg-00397/2002
A/avian/New York/Sg-00364/2001
A/avian/New York/Sg-00346/2000
A/avian/New York/Sg-00345/2000
A/avian/New York/Sg-00356/2001
A/unknown/NY/85161/2000
A/avian/New York/Sg-00366/2001
A/avian/New York/Sg-00355/2000
A/avian/New York/Sg-00359/2001
A/avian/New York/Sg-00350/2000
A/quail/New Jersey/Sg-00342/2000
A/avian/New York/Sg-00331/1999
A/chicken/FL/90348-4/2001
A/Chicken/New Jersey/30739-6/00
A/chicken/New Jersey/Sg-00340/2000
A/avian/New York/Sg-00338/1999
A/chicken/New Jersey/Sg-00336/1999
A/chicken/NY/21586-8/1999
A/unknown/New York/Sg-00337/1999
A/avian/New York/Sg-00337/1999
A/avian/New York/Sg-00339/2000
A/chicken/New Jersey/Sg-00341/2000
A/chicken/NY/30749-3/00
A/chicken/New York/Sg-00305/1998
A/chicken/New York/Sg-00306/1998
A/chicken/New York/Sg-00298/1997
A/Chicken/New York/3202-7/96
A/chicken/New York/Sg-00383/2001
A/chicken/NY/119256-7/01
A/avian/New York/Sg-00360/2001
A/avian/New York/Sg-00347/2000
A/avian/New York/Sg-00343/2000
A/avian/New York/Sg-00389/2002
A/avian/New York/Sg-00344/2000
A/avian/New York/Sg-00370/2001
A/avian/New York/Sg-00374/2001
A/avian/New York/Sg-00357/2001
A/avian/New York/Sg-00358/2001
A/avian/New York/Sg-00325/1999
A/quail/NY/13989-51/1998
A/chicken/PA/9801289/1998
A/Chicken/Pennsylvania/13552-1/98
A/turkey/PA/7975/1997
A/environment/Pennsylvania/Sg-00321/1998
A/chicken/PA/149092-1/02
A/chicken/PA/19241/1997
A/guinea fowl/New Jersey/Sg-00324/1999
A/goose/NY/8600-3/1998
A/chicken/PA/7777-1/1997
A/avian/New York/Sg-00322/1999
A/duck/New York/Sg-00310/1998
A/chicken/New Jersey/Sg-00313/1998
A/chicken/PA/11767/1997
A/guinea fowl/New Jersey/Sg-00320/1998
A/chicken/New Jersey/Sg-00314/1998
A/chicken/New Jersey/Sg-00304/1997
A/chicken/New York/Sg-00308/1998
A/guinea fowl/New York/Sg-00309/1998
A/quail/PA/20304/1998
A/chicken/New York/Sg-00288/1996
A/chicken/New Jersey/Sg-00295/1996
A/duck/New Jersey/Sg-00286/1996
A/goose/New Jersey/Sg-00290/1996
A/duck/New Jersey/Sg-00319/1998
A/turkey/New Jersey/Sg-00312/1998
A/Chicken/New York/3112-1/95
A/chicken/NY/8030-2/1997
A/chicken/New York/Sg-00281/1996
A/chicken/New York/Sg-00294/1996
A/chicken/New York/Sg-00274/1995
A/chicken/NY/13833-7/1995
A/chicken/Rhode Island/Sg-00282/1996
A/chicken/RI/4328-1/1995
A/chicken/New York/Sg-00285/1996
A/turkey/New Jersey/Sg-00287/1996
A/duck/New York/Sg-00291/1996
A/chicken/New York/Sg-00307/1998
A/guinea fowl/New Jersey/Sg-00297/1997
A/chicken/New York/Sg-00299/1997
A/avian/New York/Sg-00323/1999
A/Chicken/New York/6777-3/97
A/quail/New Jersey/Sg-00318/1998
A/avian/New York/Sg-00363/2001
A/chicken/New Jersey/Sg-00311/1998
A/shorebird/Delaware Bay/274/1994
A/chicken/New York/Sg-00270/1995
A/duck/New York/Sg-00259/1994
A/chicken/New York/Sg-00254/1994
A/Chicken/New York/13142-5/94
A/guinea fowl/New York/Sg-00257/1994
A/turkey/New York/Sg-00255/1994
A/turkey/New York/Sg-00263/1995
A/turkey/New York/4450-5/1994
A/Chicken/New York/4447-7/94
A/chicken/New York/Sg-00279/1995
A/chicken/New York/Sg-00261/1995
A/chicken/New Jersey/Sg-00292/1996
A/guinea fowl/New York/Sg-00283/1996
A/turkey/New York/Sg-00284/1996
A/chicken/New York/Sg-00277/1995
A/Chicken/New York/19542-5/95
A/chicken/New Jersey/Sg-00280/1995
A/chicken/New Jersey/Sg-00275/1995
A/turkey/NY/13820-3/1995
A/guinea fowl/New York/13820-3/1995
A/guinea fowl/New York/Sg-00271/1995
0.002

D

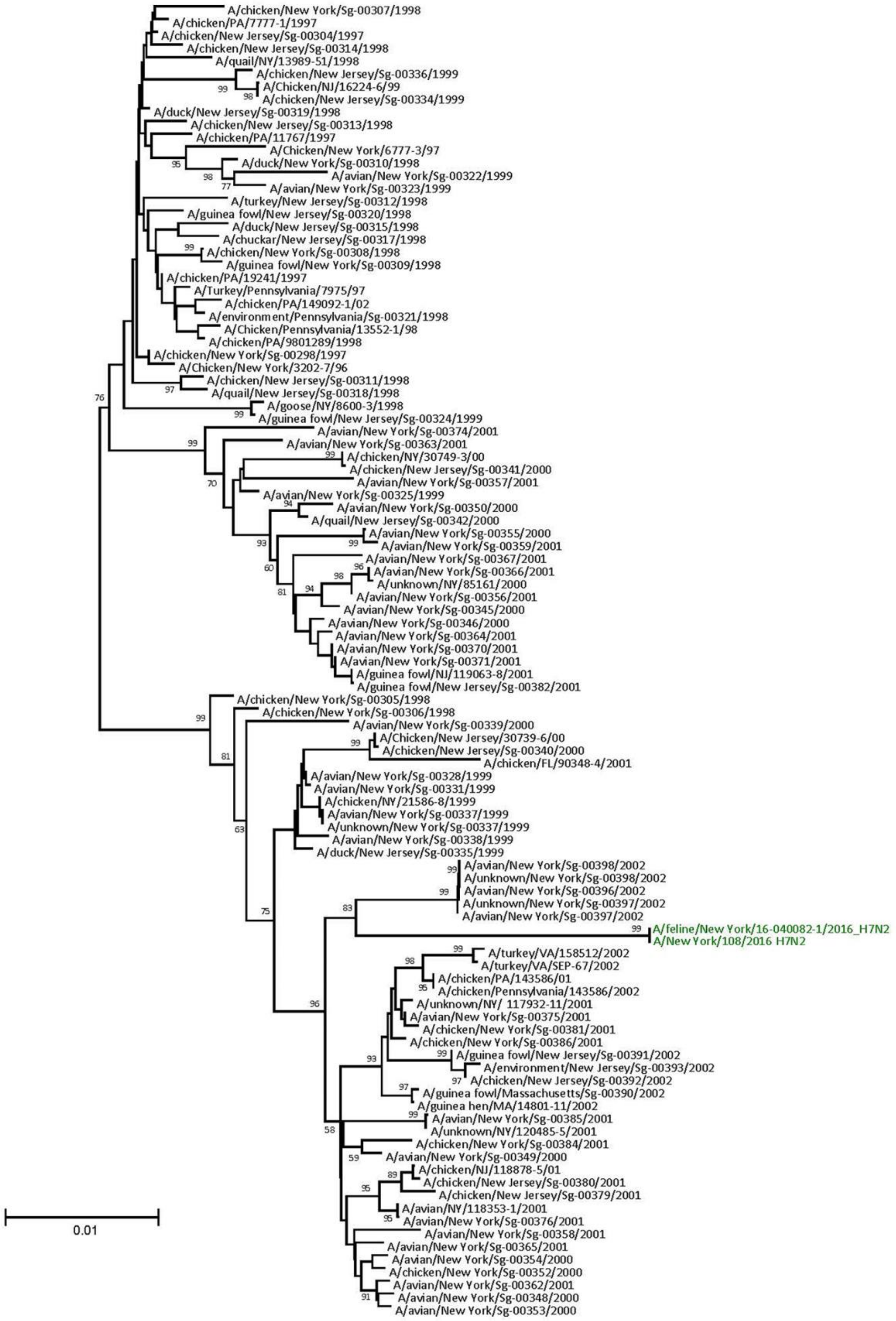
A/chicken/New York/Sg-00307/1998
A/chicken/PA/7777-1/1997
A/chicken/New Jersey/Sg-00304/1997
A/chicken/New Jersey/Sg-00314/1998
A/quail/NY/13989-51/1998
A/chicken/New Jersey/Sg-00336/1999
A/Chicken/NJ/16224-6/99
A/chicken/New Jersey/Sg-00334/1999
A/duck/New Jersey/Sg-00319/1998
A/chicken/New Jersey/Sg-00313/1998
A/chicken/PA/11767/1997
A/Chicken/New York/6777-3/97
A/duck/New York/Sg-00310/1998
A/avian/New York/Sg-00322/1999
A/avian/New York/Sg-00323/1999
A/turkey/New Jersey/Sg-00312/1998
A/guinea fowl/New Jersey/Sg-00320/1998
A/duck/New Jersey/Sg-00315/1998
A/chuckar/New Jersey/Sg-00317/1998
A/chicken/New York/Sg-00308/1998
A/guinea fowl/New York/Sg-00309/1998
A/chicken/PA/19241/1997
A/Turkey/Pennsylvania/7975/97
A/chicken/PA/149092-1/02
A/environment/Pennsylvania/Sg-00321/1998
A/Chicken/Pennsylvania/13552-1/98
A/chicken/PA/9801289/1998
A/chicken/New York/Sg-00298/1997
A/Chicken/New York/3202-7/96
A/chicken/New Jersey/Sg-00311/1998
A/quail/New Jersey/Sg-00318/1998
A/goose/NY/8600-3/1998
A/guinea fowl/New Jersey/Sg-00324/1999
A/avian/New York/Sg-00374/2001
A/avian/New York/Sg-00363/2001
A/chicken/NY/30749-3/00
A/chicken/New Jersey/Sg-00341/2000
A/avian/New York/Sg-00357/2001
A/avian/New York/Sg-00325/1999
A/avian/New York/Sg-00350/2000
A/quail/New Jersey/Sg-00342/2000
A/avian/New York/Sg-00355/2000
A/avian/New York/Sg-00359/2001
A/avian/New York/Sg-00367/2001
A/avian/New York/Sg-00366/2001
A/unknown/NY/85161/2000
A/avian/New York/Sg-00356/2001
A/avian/New York/Sg-00345/2000
A/avian/New York/Sg-00346/2000
A/avian/New York/Sg-00364/2001
A/avian/New York/Sg-00370/2001
A/avian/New York/Sg-00371/2001
A/guinea fowl/NJ/119063-8/2001
A/guinea fowl/New Jersey/Sg-00382/2001
A/chicken/New York/Sg-00305/1998
A/chicken/New York/Sg-00306/1998
A/avian/New York/Sg-00339/2000
A/Chicken/New Jersey/30739-6/00
A/chicken/New Jersey/Sg-00340/2000
A/chicken/FL/90348-4/2001
A/avian/New York/Sg-00328/1999
A/avian/New York/Sg-00331/1999
A/chicken/NY/21586-8/1999
A/avian/New York/Sg-00337/1999
A/unknown/New York/Sg-00337/1999
A/avian/New York/Sg-00338/1999
A/duck/New Jersey/Sg-00335/1999
A/avian/New York/Sg-00398/2002
A/unknown/New York/Sg-00398/2002
A/avian/New York/Sg-00396/2002
A/unknown/New York/Sg-00397/2002
A/avian/New York/Sg-00397/2002
A/feline/New York/16-040082-1/2016_H7N2
A/New York/108/2016 H7N2
A/turkey/VA/158512/2002
A/turkey/VA/SEP-67/2002
A/chicken/PA/143586/01
A/chicken/Pennsylvania/143586/2002
A/unknown/NY/ 117932-11/2001
A/avian/New York/Sg-00375/2001
A/chicken/New York/Sg-00381/2001
A/chicken/New York/Sg-00386/2001
A/guinea fowl/New Jersey/Sg-00391/2002
A/environment/New Jersey/Sg-00393/2002
A/chicken/New Jersey/Sg-00392/2002
A/guinea fowl/Massachusetts/Sg-00390/2002
A/guinea hen/MA/14801-11/2002
A/avian/New York/Sg-00385/2001
A/unknown/NY/120485-5/2001
A/chicken/New York/Sg-00384/2001
A/avian/New York/Sg-00349/2000
A/chicken/NJ/118878-5/01
A/chicken/New Jersey/Sg-00380/2001
A/chicken/New Jersey/Sg-00379/2001
A/avian/NY/118353-1/2001
A/avian/New York/Sg-00376/2001
A/avian/New York/Sg-00358/2001
A/avian/New York/Sg-00365/2001
A/avian/New York/Sg-00354/2000
A/chicken/New York/Sg-00352/2000
A/avian/New York/Sg-00362/2001
A/avian/New York/Sg-00348/2000
A/avian/New York/Sg-00353/2000
0.01

E

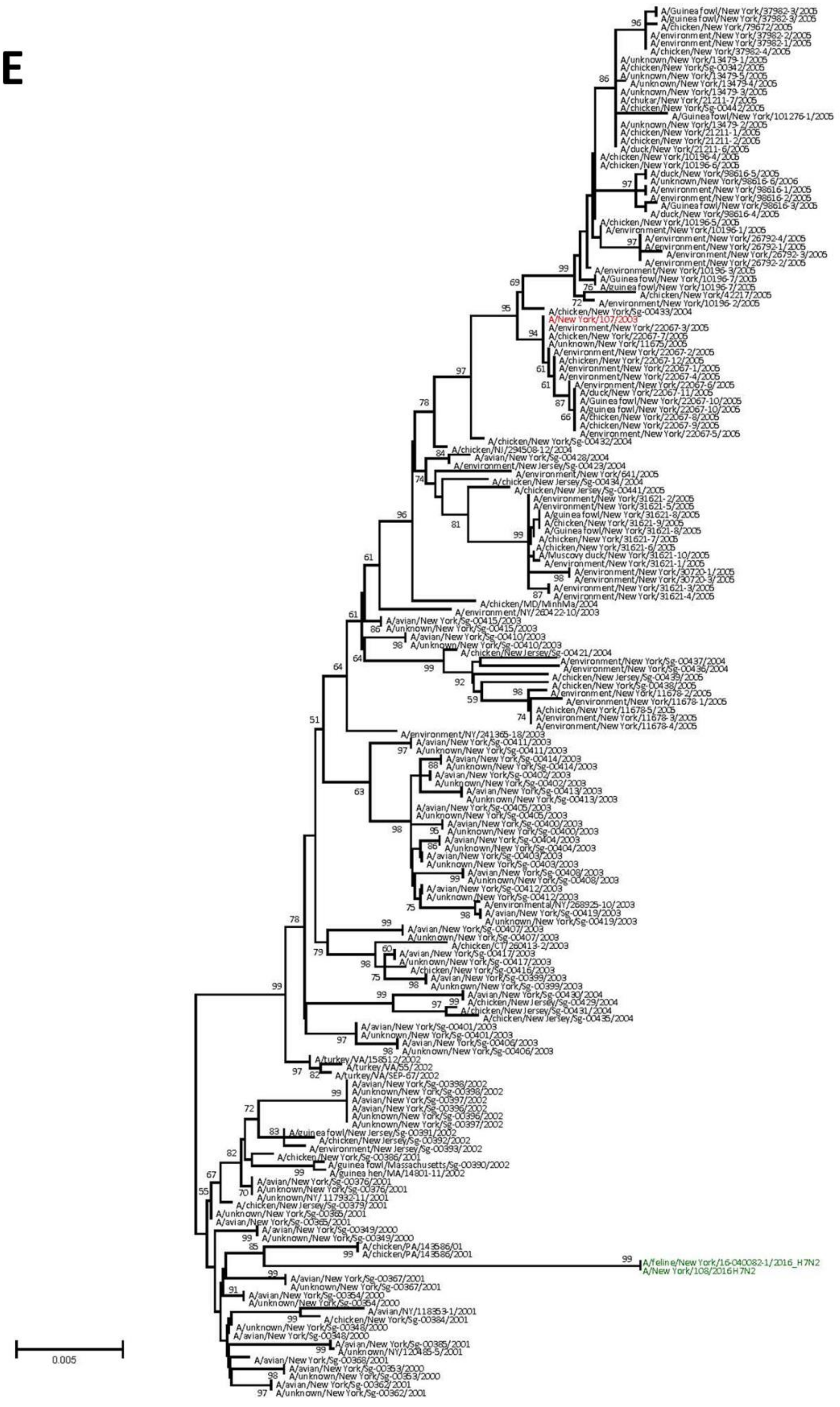

F

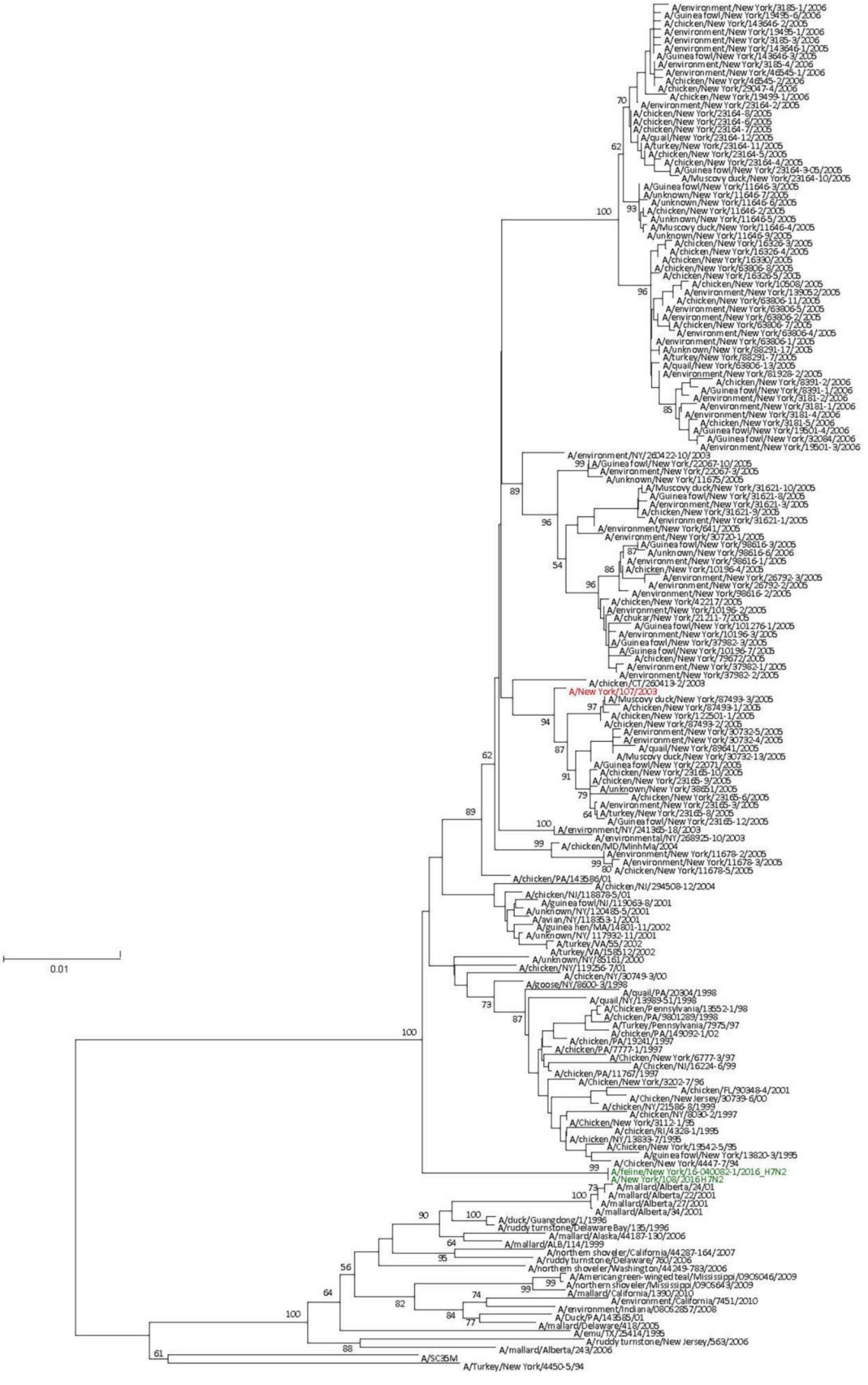

G

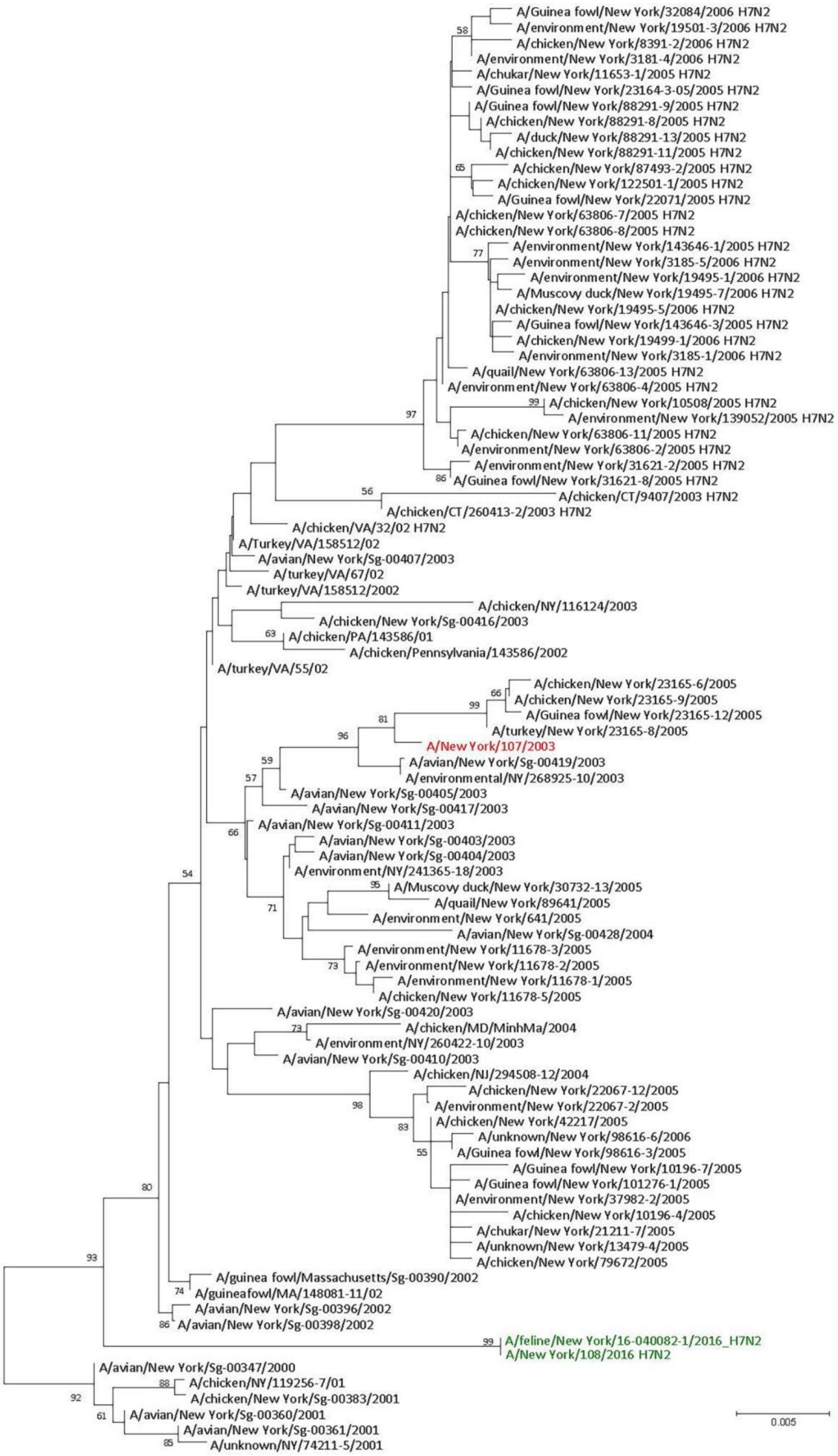

A/Guinea fowl/New York/32084/2006 H7N2
A/environment/New York/19501-3/2006 H7N2
A/chicken/New York/8391-2/2006 H7N2
A/environment/New York/3181-4/2006 H7N2
A/chukar/New York/11653-1/2005 H7N2
A/Guinea fowl/New York/23164-3-05/2005 H7N2
A/Guinea fowl/New York/88291-9/2005 H7N2
A/chicken/New York/88291-8/2005 H7N2
A/duck/New York/88291-13/2005 H7N2
A/chicken/New York/88291-11/2005 H7N2
A/chicken/New York/87493-2/2005 H7N2
A/chicken/New York/122501-1/2005 H7N2
A/Guinea fowl/New York/22071/2005 H7N2
A/chicken/New York/63806-7/2005 H7N2
A/chicken/New York/63806-8/2005 H7N2
A/environment/New York/143646-1/2005 H7N2
A/environment/New York/3185-5/2006 H7N2
A/environment/New York/19495-1/2006 H7N2
A/Muscovy duck/New York/19495-7/2006 H7N2
A/chicken/New York/19495-5/2006 H7N2
A/Guinea fowl/New York/143646-3/2005 H7N2
A/chicken/New York/19499-1/2006 H7N2
A/environment/New York/3185-1/2006 H7N2
A/quail/New York/63806-13/2005 H7N2
A/environment/New York/63806-4/2005 H7N2
A/chicken/New York/10508/2005 H7N2
A/environment/New York/139052/2005 H7N2
A/chicken/New York/63806-11/2005 H7N2
A/environment/New York/63806-2/2005 H7N2
A/environment/New York/31621-2/2005 H7N2
A/Guinea fowl/New York/31621-8/2005 H7N2
A/chicken/CT/9407/2003 H7N2
A/chicken/CT/260413-2/2003 H7N2
A/chicken/VA/32/02 H7N2
A/Turkey/VA/158512/02
A/avian/New York/Sg-00407/2003
A/turkey/VA/67/02
A/turkey/VA/158512/2002
A/chicken/NY/116124/2003
A/chicken/New York/Sg-00416/2003
A/chicken/PA/143586/01
A/chicken/Pennsylvania/143586/2002
A/turkey/VA/55/02
A/chicken/New York/23165-6/2005
A/chicken/New York/23165-9/2005
A/Guinea fowl/New York/23165-12/2005
A/turkey/New York/23165-8/2005
A/New York/107/2003
A/avian/New York/Sg-00419/2003
A/environmental/NY/268925-10/2003
A/avian/New York/Sg-00405/2003
A/avian/New York/Sg-00417/2003
A/avian/New York/Sg-00411/2003
A/avian/New York/Sg-00403/2003
A/avian/New York/Sg-00404/2003
A/environment/NY/241365-18/2003
A/Muscovy duck/New York/30732-13/2005
A/quail/New York/89641/2005
A/environment/New York/641/2005
A/avian/New York/Sg-00428/2004
A/environment/New York/11678-3/2005
A/environment/New York/11678-2/2005
A/environment/New York/11678-1/2005
A/chicken/New York/11678-5/2005
A/avian/New York/Sg-00420/2003
A/chicken/MD/MinhMa/2004
A/environment/NY/260422-10/2003
A/avian/New York/Sg-00410/2003
A/chicken/NJ/294508-12/2004
A/chicken/New York/22067-12/2005
A/environment/New York/22067-2/2005
A/chicken/New York/42217/2005
A/unknown/New York/98616-6/2006
A/Guinea fowl/New York/98616-3/2005
A/Guinea fowl/New York/10196-7/2005
A/Guinea fowl/New York/101276-1/2005
A/environment/New York/37982-2/2005
A/chicken/New York/10196-4/2005
A/chukar/New York/21211-7/2005
A/unknown/New York/13479-4/2005
A/chicken/New York/79672/2005
A/guinea fowl/Massachusetts/Sg-00390/2002
A/guineafowl/MA/148081-11/02
A/avian/New York/Sg-00396/2002
A/avian/New York/Sg-00398/2002
A/feline/New York/16-040082-1/2016_H7N2
A/New York/108/2016 H7N2
A/avian/New York/Sg-00347/2000
A/chicken/NY/119256-7/01
A/chicken/New York/Sg-00383/2001
A/avian/New York/Sg-00360/2001
A/avian/New York/Sg-00361/2001
A/unknown/NY/74211-5/2001
0.005

H

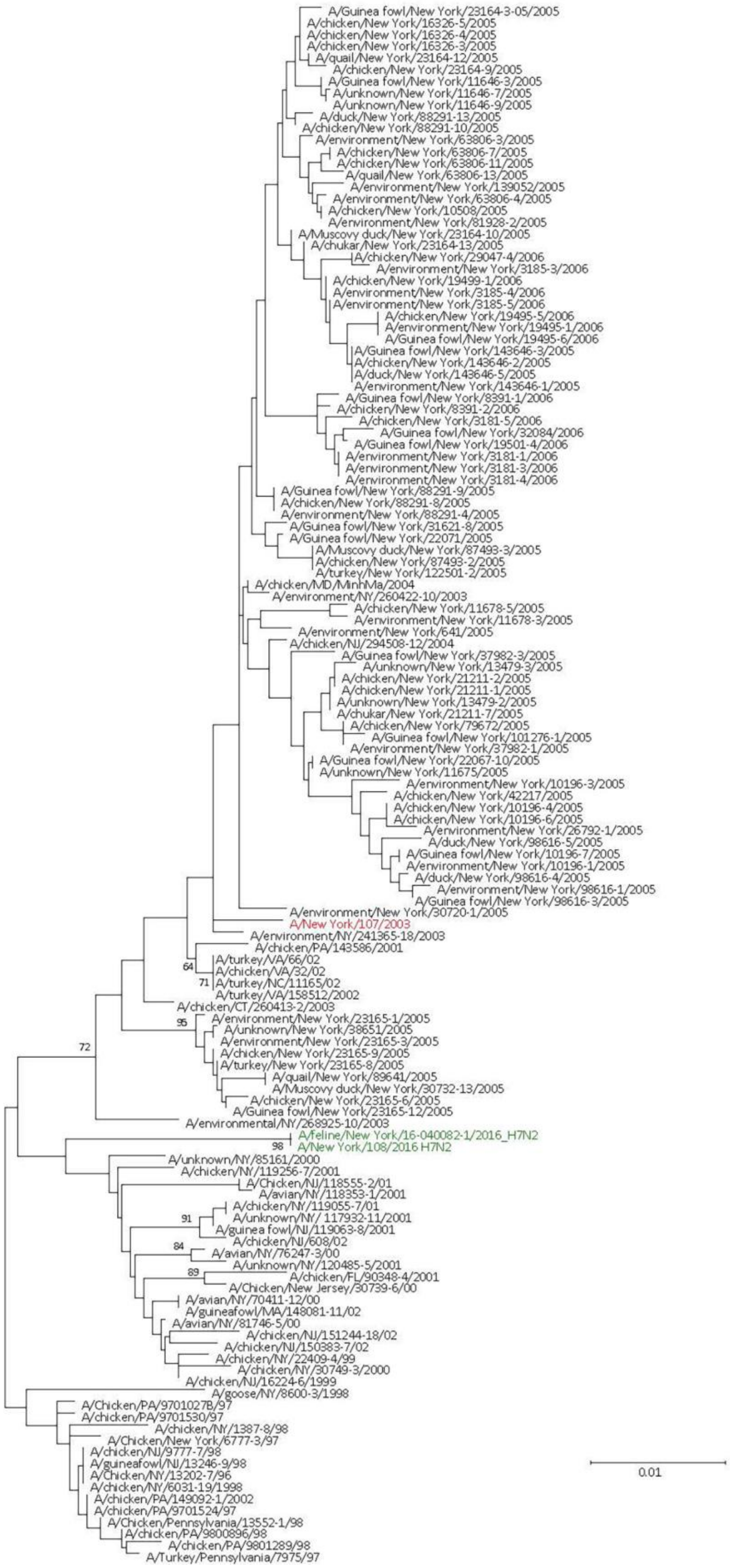
A/Guinea fowl/New York/23164-3-05/2005
A/chicken/New York/16326-5/2005
A/chicken/New York/16326-4/2005
A/chicken/New York/16326-3/2005
A/quail/New York/23164-12/2005
A/chicken/New York/23164-9/2005
A/Guinea fowl/New York/11646-3/2005
A/unknown/New York/11646-7/2005
A/unknown/New York/11646-9/2005
A/duck/New York/88291-13/2005
A/chicken/New York/88291-10/2005
A/environment/New York/63806-3/2005
A/chicken/New York/63806-7/2005
A/chicken/New York/63806-11/2005
A/quail/New York/63806-13/2005
A/environment/New York/139052/2005
A/environment/New York/63806-4/2005
A/chicken/New York/10508/2005
A/environment/New York/81928-2/2005
A/Muscovy duck/New York/23164-10/2005
A/chukar/New York/23164-13/2005
A/chicken/New York/29047-4/2006
A/environment/New York/3185-3/2006
A/chicken/New York/19499-1/2006
A/environment/New York/3185-4/2006
A/environment/New York/3185-5/2006
A/chicken/New York/19495-5/2006
A/environment/New York/19495-1/2006
A/Guinea fowl/New York/19495-6/2006
A/Guinea fowl/New York/143646-3/2005
A/chicken/New York/143646-2/2005
A/duck/New York/143646-5/2005
A/environment/New York/143646-1/2005
A/Guinea fowl/New York/8391-1/2006
A/chicken/New York/8391-2/2006
A/chicken/New York/3181-5/2006
A/Guinea fowl/New York/32084/2006
A/Guinea fowl/New York/19501-4/2006
A/environment/New York/3181-1/2006
A/environment/New York/3181-3/2006
A/environment/New York/3181-4/2006
A/Guinea fowl/New York/88291-9/2005
A/chicken/New York/88291-8/2005
A/environment/New York/88291-4/2005
A/Guinea fowl/New York/31621-8/2005
A/Guinea fowl/New York/22071/2005
A/Muscovy duck/New York/87493-3/2005
A/chicken/New York/87493-2/2005
A/turkey/New York/122501-2/2005
A/chicken/MD/MinhMa/2004
A/environment/NY/260422-10/2003
A/chicken/New York/11678-5/2005
A/environment/New York/11678-3/2005
A/environment/New York/641/2005
A/chicken/NJ/294508-12/2004
A/Guinea fowl/New York/37982-3/2005
A/unknown/New York/13479-3/2005
A/chicken/New York/21211-2/2005
A/chicken/New York/21211-1/2005
A/unknown/New York/13479-2/2005
A/chukar/New York/21211-7/2005
A/chicken/New York/79672/2005
A/Guinea fowl/New York/101276-1/2005
A/environment/New York/37982-1/2005
A/Guinea fowl/New York/22067-10/2005
A/unknown/New York/11675/2005
A/environment/New York/10196-3/2005
A/chicken/New York/42217/2005
A/chicken/New York/10196-4/2005
A/chicken/New York/10196-6/2005
A/environment/New York/26792-1/2005
A/duck/New York/98616-5/2005
A/Guinea fowl/New York/10196-7/2005
A/environment/New York/10196-1/2005
A/duck/New York/98616-4/2005
A/environment/New York/98616-1/2005
A/Guinea fowl/New York/98616-3/2005
A/environment/New York/30720-1/2005
A/New York/107/2003
A/environment/NY/241365-18/2003
A/chicken/PA/143586/2001
A/turkey/VA/66/02
64
A/chicken/VA/32/02
71
A/turkey/NC/11165/02
A/turkey/VA/158512/2002
A/chicken/CT/260413-2/2003
95
A/environment/New York/23165-1/2005
A/unknown/New York/38651/2005
A/environment/New York/23165-3/2005
72
A/chicken/New York/23165-9/2005
A/turkey/New York/23165-8/2005
A/quail/New York/89641/2005
A/Muscovy duck/New York/30732-13/2005
A/chicken/New York/23165-6/2005
A/Guinea fowl/New York/23165-12/2005
A/environmental/NY/268925-10/2003
A/feline/New York/16-040082-1/2016_H7N2
98
A/New York/108/2016 H7N2
A/unknown/NY/85161/2000
A/chicken/NY/119256-7/2001
A/Chicken/NJ/118555-2/01
A/avian/NY/118353-1/2001
A/chicken/NY/119055-7/01
91
A/unknown/NY/ 117932-11/2001
A/guinea fowl/NJ/119063-8/2001
A/chicken/NJ/608/02
84
A/avian/NY/76247-3/00
A/unknown/NY/120485-5/2001
89
A/chicken/FL/90348-4/2001
A/Chicken/New Jersey/30739-6/00
A/avian/NY/70411-12/00
A/guineafowl/MA/148081-11/02
A/avian/NY/81746-5/00
A/chicken/NJ/151244-18/02
A/chicken/NJ/150383-7/02
A/chicken/NY/22409-4/99
A/chicken/NY/30749-3/2000
A/chicken/NJ/16224-6/1999
A/goose/NY/8600-3/1998
A/Chicken/PA/9701027B/97
A/chicken/PA/9701530/97
A/chicken/NY/1387-8/98
A/Chicken/New York/6777-3/97
A/chicken/NJ/9777-7/98
A/guineafowl/NJ/13246-9/98
A/Chicken/NY/13202-7/96
A/chicken/NY/6031-19/1998
A/chicken/PA/149092-1/2002
A/chicken/PA/9701524/97
A/Chicken/Pennsylvania/13552-1/98
A/chicken/PA/9800896/98
A/chicken/PA/9801289/98
A/Turkey/Pennsylvania/7975/97
0.01

Technical Appendix Figure. Neighbor-joining phylogenetic trees of the (A) HA, (B) NA, and (C–H) internal genes. The human and feline H7N2 isolates are green. All candidate vaccine viruses are red and HI reference viruses are blue. Amino acid differences were calculated based on A/turkey/Virginia/4529/2002; bootstraps >50 generated from 1,000 replicates are shown at branch nodes. The scale bar represents nucleotide substitutions per site.
